# Small Bowel Obstruction Conservatively Managed in Hospital-At-Home

**DOI:** 10.1155/2022/1969040

**Published:** 2022-11-08

**Authors:** Margaret R. Paulson, Abdullah S. Eldaly, Francisco R. Avila, Ricardo A. Torres-Guzman, Karla C. Maita, John P. Garcia, Luiza Palmieri Serrano, Omar S. Emam, Antonio J. Forte, Michael J. Maniaci

**Affiliations:** ^1^Division of Hospital Internal Medicine, Mayo Clinic Health Systems, Eau Claire, Wisconsin 2321 Stout Road, Menomonie, Wisconsin 54751, USA; ^2^Division of Plastic Surgery, Mayo Clinic, 4500 San Pablo Road, Jacksonville, Florida 32224, USA; ^3^Division of Hospital Internal Medicine, Mayo Clinic, 4500 San Pablo Road, Jacksonville, Florida 32224, USA

## Abstract

In 2020, Mayo Clinic established an Advanced Care at Home (ACH) program. ACH is a virtual hybrid hospital-at-home (HaH) program that combines telemedicine with in-home care services by utilizing a state that is software-driven, vendor-mediate medical supply chain. The program initially focused on acute medical diagnosis but has expanded to oversee surgical and postsurgical patients with continued inpatient needs. Here, we report the first case of a small bowel obstruction (SBO) managed under a HaH program. A 52-year-old lady presented to the emergency department with symptoms suggestive of mechanical SBO. The diagnosis was confirmed with an abdominopelvic computed tomography (CT) scan, and the patient was admitted to the hospital. Based on the patient's presentation and laboratory results, the care team proceeded with conservative treatment including nasogastric tube (NG) placement and suctioning, intravenous (IV) fluid replacement, and daily laboratory studies. She spent the first hospital day in the physical hospital ward so that the surgical team could ensure stability clinically and no urgent need for surgical intervention. On hospital day two, she was transferred home with ACH where the NG suctioning and IV replacement therapy could continue, while the medical team conducted daily virtual visits to ensure continued improvement. Additionally, a paramedic and a nurse performed an in-person, head-to-toe assessment and administered medications to the patient twice daily. She spent 5 days in ACH getting acute care and then was discharged into a postacute phase equivalent to outpatient monitoring called the restorative phase. She was monitored remotely for the duration of the restorative phase for 10 more days, and then she recovered fully. This case highlights that high-acuity patients with SBO can receive invasive treatments like NG tube suction as well as be appropriately monitored for clinical decompensation by a virtual hybrid home hospital program which combines virtual care providers with an in-home vendor-mediated supply chain.

## 1. Introduction

Hospital-at-home (HaH) programs were first conceptualized by Burton and Regenstreif in 1995 as an alternative for geriatric patients requiring acute medical attention [1]. Four years later, the feasibility of the model was investigated by Leff et al. [2] in a pilot study that recruited 17 adult patients, aged 65 or older, that presented with acute conditions mandating hospitalization. The pilot study concluded that the HaH model was safe, feasible, and cost-effective [2], and the following years witnessed the successful implementation of several HaH programs across the country with satisfactory outcomes [3]. The primary motive of these programs is to reduce the problems the traditional brick-and-mortar hospitals (BAM) frequently face, particularly emergency department (ED) overcrowding and high expenses of inpatient care [3].

In 2020, Mayo Clinic established the Advance Care at Home (ACH) program. ACH combines a virtual provider-staffed command center (CC) with a smart supply chain of healthcare professionals that can deliver care to patients in the comfort of their own homes [4, 5]. The goals of this program are in accordance with Mayo Clinic's intent to provide individualized and adaptable care to the largest number of patients, including those who live in rural and underserved areas.

Since its implementation, ACH has successfully treated hundreds of patients with acute medical conditions, such as decompensated heart failure, pneumonia, and postoperative patients. We report the case of a patient who presented with a small bowel obstruction (SBO) and was successfully managed conservatively under ACH care. To the best of our knowledge, this is the first SBO case treated in a HaH program.

## 2. Case Presentation

A 52-year-old lady presented to the ED on October 26, 2021, with onset of acute epigastric pain, nausea, and ten accounts of nonbloody emesis a few hours prior to arrival. She described the pain as being sharp in nature and localized to the epigastric region near the xiphoid sternum without radiation. She denied nasal congestion, runny nose, postnasal drip, sore throat, cough, chest pain, shortness of breath, other abdominal pain, back pain, flank pain, pelvic pain, dysuria, hematuria, fever, chills, diarrhea or blood in her stool, or vomiting. She had a past medical history of recurrent small intestinal obstruction treated conservatively in 1999, February 2020, and August 2021. She additionally had a history of migraine headaches, an exploratory laparotomy done in 1986 due to a motor vehicle accident (MVA), and a lumbar spinal fracture at L3-L4 in 2017 caused by an MVA.

Her vital signs at presentation included a blood pressure of 130/72 mmHg, a heart rate of 42 beats per minute, a respiratory rate of 16 breaths per minute, an oral temperature of 36.9°C, and an SpO_2_ of 95%. The physical examination was positive for diffuse abdominal tenderness without guarding or rigidity. Abdominopelvic computed tomography (CT) scan showed SBO without evidence of free air, bowel incarceration, or abscess. Laboratory evaluation revealed leukocytosis (WBC 15.6) and an elevated lactate (2.5). The patient was admitted to the hospital where she remained in the ED due to the lack of inpatient ward bed availability. General surgery was consulted, and they advised admission to the hospital to start conservative treatment with gastric decompression, pain control, and intravenous (IV) fluid resuscitation. A nasogastric (NG) tube was placed and connected to low continuous suction. The patient was kept nil per os (NPO) and started on 0.9% normal saline IV at 100 mL/hour. She was admitted to the hospital with the expectation of 5–7 days of acute hospital care, watching for symptom resolution and the return of bowel function.

**Figure 1:**
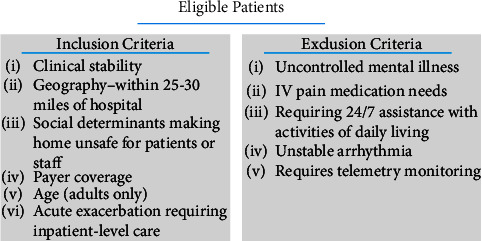
Advanced care at home patient eligibility criteria.

The following day, the patient reported that her symptoms had improved, and on examination, she had amelioration of abdominal distension and tenderness. The surgical team was encouraged that the conservative treatment was working and surgical intervention was unlikely. Although encouraged by her progress overnight, she did not want to stay an extended period of time in the BAM hospital and inquired if there were any other treatment options. The surgical team requested a consultation by the ACH team to evaluate whether the patient was a potential candidate for continuing treatment at home. The ACH team assessed the patient at bedside to determine her eligibility based on insurance, demographic, social, and clinical criteria for program admission (Figure 1) [4]. She was informed that she would be monitored very closely by a combination of both virtual and in-home personnel, and if any complications that could not be handled arose in the home, she could be rapidly transported back to the BAM hospital. At home, she would be continuously connected to the ACH Command Center (CC), which is staffed by a hospitalist physician, advanced practice providers (APPs), registered nurses (RNs), a pharmacist, and a case manager. This team would constantly assess her clinical situation and, in conjunction with the surgical team, update her medical care plan, and direct the in-home care delivery. In-home services would include APPs for advanced physical examinations, community paramedics, RNs, aides, rehabilitative services, infusion therapy, phlebotomists, and basic radiography technicians. The benefits and risks of the program were discussed in detail with the patient. Benefits included receiving hospital-level care in the comfort of her own home, called the “acute phase,” as well as ownership of her postacute care by the same care team as she recovered, called the “restorative phase.” The potential risks mentioned included delay in care due to the nature of remote management and service limitations with the use of telemedicine technology, including interruptions, unauthorized access, and technical difficulties. She was also informed that she would not be accrued any additional cost for ACH care, and here, insurance would be billed the same for both BAM and ACH care. Alternatives other than the ACH program were offered to the patient. These comprised remaining at the hospital during the acute phase of illness and seeking care from different postacute service providers after hospitalization, such as skilled nursing facilities or home health agencies. All patient questions were answered. She agreed to participate in the program and was transferred to her home on the same day.

A technological kit that consisted of a tablet for video conferencing, a two-way telephone connected to the CC and encrypted cellular cradle point, and a 72-hour backup power supply. She was also provided a personal emergency response system which consisted of a Bluetooth-connected watch with a panic button that the patient would constantly wear throughout her home hospital stay. No matter where her home location is, if any trouble arose, she would be able to activate the watch panic button which would both instantly connect her to the CC team as well as alert an in-person rapid response team. The equipment was thoroughly checked and tested for functionality. Moreover, the patient and her caregivers verbalized their understanding of how to use the equipment and when and how to contact the CC.

On her first day of the ACH acute phase (hospital day #2), the patient was contacted by her virtual bedside registered nurse (RN) to address any concerns and verify the medications and their dosages. The RN reviewed the treatment plan for the remainder of the day. Additionally, a paramedic and a nurse visited the patient twice daily to administer medications and perform complete assessment of the cardiac, gastrointestinal, genitourinary, psychosocial, musculoskeletal, peripheral vascular, and respiratory systems. At that time, the patient was kept NPO and had an NG tube maintained on a home low intermittent suctioning device. The patient received the following medications while at home: acetaminophen suppositories 325 mg, D5W and NaCl 0.45% with KCl 20 mEq/L infusion, enoxaparin injection 40 mg, ketorolac injection 15 mg, lidocaine 5% 1 patch, ondansetron injection 4 mg, ondansetron tablet 4 mg, and pantoprazole injection 40 mg.

On the third hospital day, the general surgery team made their virtual rounds on the patient. The patient reported 2/10 abdominal pain, no nausea, and had started to pass flatus. The surgical team recommended clamping the NG tube for several hours and then conducting a Gastrografin contrast enhanced abdominal radiograph series. As this advanced imaging could not be done in the home, the patient was informed that the ACH standard of care was to transport her back to the BAM hospital in a monitored medical ambulance directly to the radiology department, bypassing the ED or the hospital wards, and have the imaging study completed. Her inpatient status would remain the same, just as a patient in the BAM receiving a radiological study, and there would be no additional cost for this procedure. After completion, she was transported back home and awaited the results. The study revealed passage of contrast from the small bowl to the colon. Later that day, the RN checked the NG tube residual, which was 5 mL after 4 hours of clamping, and the NG tube was then removed.

On hospital day 4, the patient was started on small sips of clear fluids, which she tolerated. No abdominal pain, distension, nausea, or vomiting was reported by the patient to the virtual teams. She was encouraged to start ambulating more in her home to prevent deconditioning. The following day she had her first bowel movement and denied any abdominal pain or discomfort. Her diet was advanced to a full liquid diet per her request. The IV fluids were reduced from 100 mL/hr to 75 mL/hr.

On her fifth day of the ACH acute phase (hospital day #6), no pain, nausea, or vomiting was documented. She reported as feeling quite well, and she was advanced to a low fiber diet. It was decided by the ACH team that she was well enough to be discharged from the hospital and was moved to the postacute care, restorative phase of ACH. The IV fluids were stopped, the IV access was removed, and her medications were changed to oral administration.

**Figure 2:**
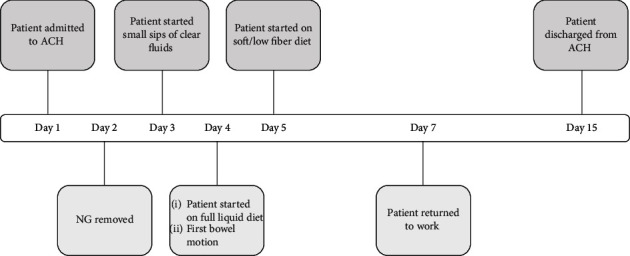
Timeline showing 5 days in the acute phase of inpatient-equivalent care and 10 days in the restorative phase of postacute recovery care.

During the posthospital restorative phase, the patient received a video call every morning from an RN to perform an assessment, review her vital data, and confirm the medications. Additionally, the patient was requested to call the CC twice daily to report her vitals and any updates related to her condition. She had a virtual visit with a physical therapist who instructed her on exercises she could do at home to improve strength and mobility. Two days after the discharge from the acute hospital phase, the patient returned to work. She was monitored for additional 8 days in the restorative phase, and upon advancing to a regular diet and having an uneventful treatment course, she was discharged from this postacute monitoring phase. The total care time was 16 days, with 1 day in the BAM hospital, 5 days in the hospital-level ACH acute phase, and 10 days in the postacute restorative phase (Figure 2).

## 3. Discussion

Telemedicine and HaH programs are effective in reducing costs of medical services, in-hospital admission, and readmission rates while improving outcomes and patient satisfaction [6–8]. The potential of these programs has escalated due to the exponential growth of technology in the last decade. Here, we present a patient with a SBO treated with conservative management in our ACH program. The patient was treated in both the acute and postacute phase and was able to avoid a prolonged stay in an inpatient ward. This is important as SBO has traditionally been seen as a condition that was only able to be treated in a BAM hospital setting. By combining virtual telemedicine with in-home, high-acuity care, we were able to accomplish treatment and recovery in an alternative setting.

SBO comprises 4% and 16% of abdominal pain related ED visits and surgical admissions in the US, respectively [9, 10]. These admissions alone result in over 2 billion USD costs annually in the US [10–12]. Postoperative adhesions account for 80% of SBO cases and are associated with higher recurrence rates [10]. Conservative management is the conventional treatment for patients with uncomplicated SBO and is successful in 43–73% of patients. Moreover, most patients show signs of clinical improvement within the first 48 hours [13–15]. IV fluid resuscitation, analgesics, antiemetics, and bowel rest constitute the conservative management of SBO [10]. All these measures can be easily delivered with a HaH program. However, the challenge in managing a patient with SBO at home is the continuous monitoring for signs of clinical deterioration (e.g., fever, leukocytosis, and tachycardia) to permit a timely surgical intervention, if needed, and prevent any compromise to the patient's outcome.

We believe that this challenge can be appropriately addressed by the ACH program as it permits real-time, hospital-level monitoring of patients due to synchronous telemedicine with standard HaH services and allows timely interventions when necessary. The patients are observed in their homes using biometric devices that collect the vital data that are normally gathered in the hospital. Furthermore, a two-way telephone permits 24/7 communication between patients, and the medical team and the emergency response system bracelet keeps patients connected to the care team no matter where the location of the home is. This process of synchronous telemonitoring is enabled by the CC where RNs, physician assistants, and advance nurse practitioners collaborate with nonclinical service coordinators to ensure the potency and quality of the service. Additionally, the ACH program is equipped with a highly qualified care team, including nurses, paramedics, phlebotomists, radiology technicians, and oxygen services. This group permits any changes in the management plan to take place smoothly in a time-efficient fashion. Finally, advanced, nontraditional in-home interventions like an NG tube connected to low intermittent suction were able to be performed. The clinical team did not start with such interventions at the onset of ACH in 2020, but as the vendor-mediated supply chain and in-home rapid response system grew and was repeatedly proven to perform at a high level, more advanced treatment options such as the above were added to expand ACH use cases. New interventions require elaborate planning for in-home vendor redundancy in order to meet safety guidelines as well as to provide the opportunity for education on these new treatment modalities for both the in-home vendor and the virtual providers. Often, simulation exercises are performed prior to implementation in real patient care in order to make sure all the components work as expected and the ACH team is satisfied with the quality, safety, and experience.

The second key factor in this case was the lack of inpatient ward bed availability, which resulted in the patient being kept in the ED for 24 hours prior to moving her to ACH. The lack of bed availability likely played a role in the patient's decision to enroll into the ACH program. Many hospital systems suffer from the lack of bed capacity, resulting in patients who should be receiving care in an inpatient ward to instead receive ward-level care in the ED where the patient is boarded [16, 17]. This leads to ED overcrowding, which increases morbidity and mortality for both boarded and ED patients as well as increases the length of stay of the admitted patients leading to decreased patient and staff satisfaction [16]. One solution to this problem is the creation of virtual wards through HaH programs. HaH programs can provide much needed hospital capacity creation and serve as a relief valve for overcrowded facilities. This capacity creation was seen many times during the COVID-19 pandemic in 2020–2022, with many institutions using the HaH model to reduce their BAM admissions [18]. Although ACH was used to manage some postoperative patients who required continuous monitoring, this was the first time an acute SBO was admitted into the program. We did not initially start our ACH program with patients who need complex in-home interventions like NG tube suction and monitoring for bowel perforation and peritonitis. But the growth of our rapid response system and supplier network resulted in our virtual care providers to feel much more comfortable to treat high-acuity surgical patients like SBO in ACH. Once confident in this new mode of care delivery, our ACH team engaged our inpatient surgical colleagues in order to solidify workflows and communication chains so that we could partner in enrolling SBO patients in ACH. Due to this process and partnership, our patient was transferred smoothly from the BAM hospital to ACH, received hospital-level care at her home, and was discharged with a favorable outcome. Furthermore, she returned to work while being monitored by the ACH staff, which is an advantage that could not be achieved under the usual BAM care.

## 4. Conclusion

This case shows that patients who may need surgical intervention due to a high-acuity diagnosis like SBO can be safely monitored in a hospital-at-home program and receive inpatient-level care including NG suction, laboratory studies, and IV fluids. This case is an example of home building the proper in-home care delivery system overseen by virtual care providers which can offer an alternative care setting in the treatment of SBO. This can have the added benefits of both reducing brick-and-mortar admissions, resulting in improved hospital capacity, and providing the patient with the more comfortable experience of recovering from their illness in their own home. Our hospital will continue to look at ACH as the primary choice in care setting for clinically stable SBO patients. We will continue to conduct studies to discover what other surgical diagnosis can be safely treated inhome hospital.

## Data Availability

The data generated during and/or analyzed during the current study are available from the corresponding author on reasonable request..
